# Epithelial to mesenchymal transition and microRNA expression are associated with spindle and apocrine cell morphology in triple-negative breast cancer

**DOI:** 10.1038/s41598-021-84350-2

**Published:** 2021-03-04

**Authors:** Marketa Koleckova, Jiri Ehrmann, Jan Bouchal, Maria Janikova, Aneta Brisudova, Josef Srovnal, Katerina Staffova, Marek Svoboda, Ondrej Slaby, Lenka Radova, Katherine Vomackova, Bohuslav Melichar, Lucia Veverkova, Zdenek Kolar

**Affiliations:** 1 Department of Clinical and Molecular Pathology, Faculty of Medicine and Dentistry, Palacky University and University Hospital, 775 15 Olomouc, Czech Republic; 2 Institute of Molecular and Translational Medicine, Faculty of Medicine and Dentistry, Palacky University and University Hospital, 775 15 Olomouc, Czech Republic; 3 Central European Institute of Technology, Masaryk University, 625 00 Brno, Czech Republic; 4 Department of Surgery I, Faculty of Medicine and Dentistry, Palacky University and University Hospital, 775 15 Olomouc, Czech Republic; 5 Department of Oncology, Faculty of Medicine and Dentistry, Palacky University and University Hospital, 775 15 Olomouc, Czech Republic; 6 Department of Radiology, Faculty of Medicine and Dentistry, Palacky University and University Hospital, 775 15 Olomouc, Czech Republic

**Keywords:** Breast cancer, Cancer genetics, Cancer microenvironment

## Abstract

Triple negative breast cancers (TNBC) are a morphologically and genetically heterogeneous group of breast cancers with uncertain prediction of biological behavior and response to therapy. Epithelial to mesenchymal transition (EMT) is a dynamic process characterized by loss of typical epithelial phenotype and acquisition of mesenchymal characteristics. Aberrant activation of EMT can aggravate the prognosis of patients with cancer, however, the mechanisms of EMT and role of microRNAs (miRNAs) in EMT activation is still unclear. The aim of our study was to analyze miRNA expression within areas of TNBCs with cellular morphology that may be related to the EMT process and discuss possible associations. Out of all 3953 re-examined breast cancers, 460 breast cancers were diagnosed as TNBC (11.64%). With regard to complete tumor morphology preservation, the tissue samples obtained from core—cut biopsies and influenced by previous neoadjuvant therapy were excluded. We assembled a set of selected 25 cases to determine miRNA expression levels in relation to present focal spindle cell and apocrine cell morphology within individual TNBCs. We used descriptive (histological typing and morphology), morphometric, molecular (microdissection of tumor and non-tumor morphologies, RNA isolation and purification, microchip analysis) and bioinformatic analysis (including pathway analysis). The results were verified by quantitative real-time PCR (RT-qPCR) on an extended set of 70 TNBCs. The majority of TNBCs were represented by high—grade invasive carcinomas of no special type (NST) with medullary features characterized by well-circumscribed tumors with central necrosis or fibrosis and frequent tendency to spindle-cell and/or apocrine cell transformation. Apocrine and spindle cell transformation showed a specific miRNA expression profile in comparison to other tumor parts, in situ carcinoma or non-tumor structures, particularly down-regulated expression of hsa-miRNA-143-3p and hsa-miRNA-205-5p and up-regulated expression of hsa-miR-22-3p, hsa-miRNA-185-5p, and hsa-miR-4443. Apocrine cell tumor morphology further revealed decreased expression of hsa-miR-145-5p and increased expression of additional 14 miRNAs (e.g. hsa-miR-182-5p, hsa-miR-3135b and hsa-miR-4417). Pathway analysis for target genes of these miRNAs revealed several shared biological processes (i.e. Wnt signaling, ErbB signaling, MAPK signaling, endocytosis and axon guidance), which may in part contribute to the EMT and tumor progression. We provide the first miRNA expression profiling of specific tissue morphologies in TNBC. Our results demonstrate a specific miRNA expression profile of apocrine and spindle cell morphology which can exhibit a certain similarity with the EMT process and may also be relevant for prognosis and therapy resistance of TNBC.

## Introduction

Epithelial to mesenchymal transition (EMT) is a dynamic process characterized by reversible conversion of a polarized epithelial cell to a mesenchymal cell phenotype. EMT plays a pivotal role in embryogenesis and organ morphogenesis (type I), tissue regeneration and repair (type II) and tumor progression with metastatic spread of primary epithelial tumors and/or treatment resistance (type III)^1–3^. The aberrant activation of EMT promotes the loss of typical epithelial characteristics (tight intercellular junctions, apical-basal cell polarity with relatively uniform structural arrangement, rigidity of the extracellular matrix) and leads to acquisition of mesenchymal attributions, such as enhanced migratory capacity, invasiveness, increased resistance to apoptosis and elevated production of extracellular matrix (ECM) components (Table 1). EMT can be induced by several signaling pathways including TGF—β1, Notch, Wnt and GSK3β^4^. EMT- related cell transformation is accompanied by distinct molecular processes, such as activation of transcription factors (SNAIL1/2,bHLH—E47 and E2-2, TWIST1/2, KLF8, PRRX1, FOXC2, GSC, LBX1), alteration in expression of specific cell surface, cytoskeletal and nuclear proteins, degradation of basal cell membrane, differential splicing, translation and posttranslational control^5,6^. These attributes are accompanied by a change in cell morphology including spindle cell or apocrine transformation^7,8^. The role of miRNAs in these processes is still unclear. The plasticity of epithelial phenotype enables the repetitive reverse conversion—mesenchymal to epithelial transition (MET) and metastatic spread of tumor cells. EMT is also implicated in chemoresistance, tumor recurrence and induction of cancer stem cell (CSC) self-renewal and differentiation.

**Table 1:** Morphological and molecular characteristics of EMT.

Cellular processes involved in EMT	Characteristic changes	References
Tumor cell transformations	O spindle-cell, apocrine and giant cells (mesenchymal patterns)	^10^
Structural changes of cytoskeleton	O structural reorganization, lack of cytokeratin expression and apical to basal polarity	^1–8^
Aggressive tumor biological behavior	O tumor cell proliferation, migration, invasion, extravasation and metastazing	^1–8^
Resistance to apoptosis	O altered signalling pathway WNT/β-catenin, TGF-β, TNF-α, FOXO3A, CYLD, PTEN, PFN1, RIP1,DR4/DR5, XIAP, CD147, Bcl-2, PKCε, SOCS1, cIAP1	^24^
Cancer-associated metabolic reprogramming	O enhanced glycolysis, pentose phosphate pathways, glutamine metabolism	^3,4,25^
	O biosynthesis of amino acids, nucleotides and lipids	^3,4,25^
Regulation of transcription factors and oncogenic signalling pathways	O activation of TGF-β, ZEB1, Pygo2/Wnt, STAT3, EGF, EpCAM, p-Akt S473, MCT-1/miR-34a/IL-6/IL-6, KRT19, AKT2, CD24, TIMP1	^1–8^
	O inhibition of MMP2, ALDH1, MMP9, TWIST, NOTCH1, AKT1	^1–8^
Altered expression of cell surface, cytoskeletal and nuclear proteins Changes in miRNA (miR) expression	O induced expression of N-cadherin, vimentin, desmin, α-SMA, FSP1, CDH11, β-catenin, osteonectin, fibronectin, MMP-2, MMP-3, MMP-9, Sox10, Goosecoid, Twist, Snail1, Snail2, Smad-2/3, NF-kβ	^1–8^
	O reduced expression of E-cadherin, desmoplakin, cytokeratins, occludin	^1–8^
	O increased activity of Rho, ILK, GSK-3β	^1–8^
	O **increased expression** of miR-let-7f.-5p, miR-9, miR-10a-5p, miR-10b, miR-21, miR – 29a, miR-30b, miR-30d, miR-34a, miR-93, miR-99a, miR–100, miR-101–1, miR-101-3p,miR-106, miR – 125b, miR-130a-3p,miR-155, miR-181a,miR-182-5p, miR-192-5p, miR-214-3p, miR-221, miR-222, miR-373, miR-455-3p, miR-4800-3p, miR-6836-3p, miR-4443; miR-7110-5p; miR-1225-5p; miR-885-3p; miR-22-3p	^30,36–81^
	O **reduced expression** of miR-7-5p,miR- let-7c, miR-15b, miR-17-5p, miR-20b-5p, miR-26b-5p, miR-30f. , miR-107, miR-128, miR-138-5p, miR-145-5p, miR-146a-5p, miRNA-149-3p, miRNA-150-5p, miR-186-5p, miR-190,miR-200a, miR-200b, miR-200c, miR-200c/141 cluster, miR-203, miRNA-205-5p, miR-206, miR-215, miR-223-3p,miR-335,miR-339-5p, miR-363-5p, miR-375, miR-425, miR-516a-3p, miR-520a-3p, miR-520c,miR-4324, miR-4655-3p, miR-4784, miR-6787-5p, miR-143-3p; miR-145-5p; miR-185-5p	^30,36–81^
Epithelial—mesenchymal plasticity	O MET and metastases formation	^2^

Triple-negative breast cancer (TNBC) is a molecular subtype accounting for 12–18% of all invasive breast cancers. It is determined by low or negative hormone receptor (estrogen and progesterone receptor) and HER2 protein expression, as well as *HER2* gene amplification. The occurrence of TNBC is predominantly associated with younger age at the time of diagnosis (women less than 50 years old), and genetic alterations (*BRCA1*, *TP53, PIK3CA* and *MYC* mutation). TNBCs are mostly represented by high-grade invasive carcinomas of no special type (NST), followed by metaplastic carcinomas^9^. Nevertheless, TNBCs are considered as genetically and morphologically heterogeneous group with aggressive biological behavior and specific response to therapy^10–12^. The genomic and molecular basis of adaptive or acquired chemoresistance remains poorly understood. The recent cluster analysis revealed the unique genetic expression profiles of some TNBC subtypes displaying high levels of genomic instability and enrichment for genes related to EMT and immune cell infiltration^12–14^. It has been reported that poorly differentiated tumors display a reduced expression of total miRNA. Targeting of EMT modulators implicated in proteins and enzymes of miRNA biosynthesis constitutes the most common regulatory mechanism of epithelial plasticity and metastatic tumor progression.

Many deregulated oncogenic and tumor suppressor miRNAs are associated with TNBC initiation, progression, EMT and metastatic process (See Tables 4, 5). miRNAs are also involved in cell cycle regulation, cell proliferation, apoptosis and resistance to chemotherapy. With regard to their stability in circulation, they are considered to be new diagnostic, prognostic and predictive biomarkers^15–17^.

In comparison to genetic alterations, epigenetic modifications are for the most part enzymatic. The potential reversibility of these changes suggests them as promising targets for future therapy. They involve DNA methylation, modification of histones or miRNA expression with corresponding protein synthesis pattern. Deregulation of miRNA expression results in tumor progression and metastatic spread in part by aberrant activation of EMT and resistance to therapy^18^. Understanding the regulation of EMT by miRNAs deepens our knowledge about TNBC pathogenesis with impact on overall patient survival.

## Methods

The aim of our study was to investigate the specific miRNA expression levels in TNBC cells with apocrine and spindle cell morphology in context of their function as EMT regulators. For these purposes we used descriptive (histological typing and morphology), molecular (microdissection of tumor and non-tumor morphologies, microarray and RT-qPCR analysis) and bioinformatic analysis.

### Set of patients

In our study we re-examined 3953 breast cancers investigated in the Department of Clinical and Molecular Pathology, Faculty of Medicine and Dentistry, Palacky University and University Hospital Olomouc between 2007 and 2018. Using standard immunohistochemistry (IHC), we found 460 breast cancers diagnosed as triple negative molecular subtype. We assembled a set of selected 25 cases to determine miRNA expression levels in relation to present focal spindle cell and apocrine cell morphology within individual TNBCs. We excluded all tumors which were not complete (core-cut biopsies) and which were treated by previous neoadjuvant chemotherapy. Thus, the cohort of tumors were of limited size, varied from 5 to 20 mm (pathological tumor stage pT1a–pT1c), with an average size of 13.4 mm (median 15 mm). Despite the triple negative phenotype, we also excluded the histological tumor types associated with favourable prognosis (adenoid cystic carcinomas). We also re-classified all tumors according to the latest (2019, 5th edition) WHO classification^19^ and analyzed them via descriptive, molecular biological methods and bioinformatics. To determine miRNA expression levels by Affymetrix expression profiling, we assembled a set of selected 25 cases which contained multiple tumor morphologies, particularly focal spindle cell and apocrine cell areas (Supplementary Table 1a). For RT-qPCR validation we used the set of 70 TNBCs (Supplementary Table 5), including newly microdissected tissue areas from 14 patients which were previously analysed by the Affymetrix microarrays. In total, 81 TNBC patients were used for microdissection and their joint characteristics is provided at the beginning of the Results section.

The mean age of the patients was 57.3 years (range from 28 to 82 years, median 59 years). Metastatic involvement of regional axillary lymph nodes was demonstrated in 14 cases (14/81; 17.3%). Multifocal tumor incidence was found in 8 cases (8/81; 9.9%).

### Descriptive methods

In all cases we revised the tumor histological type, tumor morphology, tumor grade and stage in the context with the age of patients at the time of diagnosis. The occurrence of central tumor necrosis/fibrosis, foci of specific tumor cell transformation, presence of in situ carcinoma was studied. The standard IHC was performed on 5 µm breast cancer formalin-fixed, paraffin-embedded (FFPE) sections. The sections were incubated with diluted primary antibodies (Table 2). The absence of staining when the primary antibody was omitted was considered as a control for nonspecific binding of the secondary antibody.

**Table 2:** Primary antibodies used in immunohistochemistry.

Biomarker	Type of primary antibody	Manufacturer	Concentration
ER	Mouse, monoclonal, 1D5	Dako; Agilent Technologies, Inc	1:20
PR	Mouse, monoclonal, PgR 636	Dako; Agilent Technologies, Inc	1:100
HER2	Rabbit, polyclonal, HercepTest™	Dako; Agilent Technologies, Inc	RTU
Vimentin	Mouse, monoclonal, V9	Dako; Agilent Technologies, Inc	1:10
E-cadherin	Rabbit, polyclonal,NCH-38	Dako; Agilent Technologies, Inc	1:50
GCDFP-15	Mouse, monoclonal, 23A3	Leica Biosystems	1:40

*RTU* ready to use.

### Molecular biological methods

In order to examine specific tumor (spindle cell, apocrine cell, ductal in situ carcinoma—DCIS, invasive front of tumor) and non-tumor areas (normal ducts and lobules, tumor-infiltrating lymphocytes—TILs) of TNBC we used PALM MicroBeam laser capture microdissection (LCM) with PALM Robo Software version 4.6 (Cat. No. 415109–2620-102; Carl Zeiss Microscopy GmbH, Jena, Germany). The procedure was performed on 10 µm breast cancer sections mounted onto 6 glass slides with RNAse-free conditions. After mounting, the slides were dried overnight in a drying oven at 56 °C and deparaffinized. For high quality RNA we used freshly prepared and precooled staining solution of Cresyl Violet.

Total RNA including small RNA was purified from microdissected FFPE tumor samples using the All Prep DNA/RNA FFPE kit (Qiagen, Hilden, Germany) according to the manufacturer’s instructions. Deparaffinization steps weren’t done due to the minimal content of paraffin in microdissected tissues. Incubation with Proteinase K at 56 °C was prolonged to 1 h instead of 15 min. RNA concentration and purity was assessed using Nanodrop ND 1000 (ThermoScientific, Wilmington, DE, USA). The RNA quality was measured by Bioanalyzer 2100 (Agilent) with Agilent Small RNA kit. Microarray analysis of 2578 miRNAs was performed by MiRNA 4.0 Array and FlashTagTM Biotin HSR RNA Labeling Kit (Applied Biosystems, Foster City, CA, USA) with use of 130 ng of total RNA.

Candidate miRNAs (hsa-miR-143-3p and hsa-miR-205-5p as downregulated in apocrine and spindle cell morphology; hsa-miR-182-5p and hsa-miR-4417 as upregulated in apocrine morphology; hsa-miR-185-5p as upregulated in both morphologies; hsa-miR-155-5p as upregulated in spindle cell morphology and tumors with lymphocytic infiltration) were quantified using miRCURY LNA miRNA PCR System (Qiagen) on the set of 70 TNBC cases (Supplementary Table 5). Complementary DNA (cDNA) was synthesized from total RNA using miRCURY LNA RT Kit (Qiagen). Amplifications were performed by miRCURY LNA SYBR Green PCR Kit (Qiagen) and specific miRCURY LNA miRNA PCR Assays (Qiagen) in triplicate on a LightCycler 480 instrument. For the intercalating green dye chemistry, RT-qPCR protocol consisted of a denaturation step at 95 °C for 2 min, followed by 40 amplification cycles at 95 °C for 5 s and 60 °C for 20 s. Melting curve analysis was performed according to the manufacturer´s protocol. For the probe-detection technology, RT-qPCR protocol consisted of a denaturation step at 95 °C for 3 min, followed by 45 amplification cycles at 95 °C for 10 s and 60 °C for 25 s. Spike-in RNA was used as endogenous control for RT-qPCR procedure. U6 was considered to be a reference gene.

### Bioinformatics

For the microarray analysis, the resulting CEL files were read and processed by R, ver. 3.5.0 (2018–04-23) (R Core Team, 2018). Data are available at the Gene Expression Omnibus under the accession number GSE162670. The linear models for microarrays (limma) were applied for comparison of resulting miRNA expression in tumor and non-tumor morphologies mentioned above. We considered the Benjamini–Hochberg adjusted p-value less than 0.05 to be statistically significant. MiRNA expression profiles of microdissected morphologies (Supplementary Table 1a) were compared by both paired and unpaired analysis. Selection of 20 miRNAs for Table 3 was based primarily on paired analysis, nevertheless 15 of them were found by unpaired analysis, too (Supplementary Tables 1b and 1c, respectively).

**Table 3:** Significantly changed levels of miRNAs in selected tumor morphologies.

miRNA	Invasive	Apocrine	CIS	Spindle	AveExpr	adj.P.Val
	log2FC	log2FC	log2FC	log2FC		
hsa-miR-143-3p	− 2.8	− 3.1	− 4.1	− 2.1	4.03	0.000
hsa-miR-885-3p	0.6	2.0	0.2	1.6	2.70	0.000
hsa-miR-3687	0.9	2.6	0.2	0.9	2,30	0.000
hsa-miR-145-5p	− 2.9	− 3.4	− 3.8	− 1.9	5.29	0.003
hsa-miR-205-5p	− 3.2	− 3.9	− 3.8	− 4.3	4.70	0.004
hsa-miR-3937	0.4	2.1	− 0.4	1.4	2.68	0.004
hsa-miR-4417	0.5	2.8	− 0.4	0.2	2.84	0.004
hsa-miR-4443	1.9	2.9	1.7	2.6	5.34	0.009
hsa-miR-3135b	0.9	2.8	− 0.1	1.8	3.50	0.012
hsa-miR-1268a	0.9	2.1	0.4	1.9	4.67	0.013
hsa-miR-22-3p	0.9	2.2	− 0.2	2.4	3.18	0.015
hsa-miR-1231	0.3	2.1	− 0.1	1.2	2.89	0.020
hsa-miR-185-5p	0.9	2.1	0.7	2.5	3.26	0.023
hsa-miR-3648	0.6	2.1	0.5	1.1	2.81	0.023
hsa-miR-574-3p	0.5	2.3	− 0.3	1.6	3.17	0.025
hsa-miR-6802-5p	0.6	2.1	0.5	1.9	2.88	0.031
hsa-miR-7110-5p	0.5	2.3	0.5	1.8	2.93	0.031
hsa-miR-182-5p	2.2	3.3	1.5	1.5	3.48	0.038
hsa-miR-1225-5p	0.3	2.0	0.1	0.6	2.87	0.038
hsa-miR-4632-5p	0.6	2.2	1.1	1.6	4.91	0.048

*AveExpr* average miRNA expression calculated from all samples, *log2FC* log2 of fold change miRNA expression in the respective tumor morphology in comparison to the normal breast epithelium.

We have also performed pathway analysis by KEGG and Gene Ontology resources. Target genes of candidate miRNAs were defined by miRDB^20^ or TargetScan^21^ databases. Pathway analyzes were performed by the KEGGprofile^22^ and clusterProfiler^23^ R-packages.

For the RT-qPCR analysis, relative quantification was carried out according to the ΔCt method using a reference gene (ΔCt = Ct target miRNA – Ct reference U6) and inverse values of ΔCt (-ΔCt) were used for subsequent statistical analysis and visualization. For the direct comparison of microarray and RT-qPCR results from the same RNA samples, a transformation to the value of 40 was performed (ΔCt = 40 − Ct target miRNA). The data were analyzed using the Spearmann correlation coefficient, Kruskal–Wallis and Mann–Whitney tests in program Statistica 12 (TIBCO Software Inc.).

### Ethics approval and consent to participate

The study was approved by the Ethics Committee of the University Hospital Olomouc and Medical Faculty of Palacky University in compliance with the Helsinki Declaration (Ref No. 111/15). According to the Czech Law (Act. No. 373/11, and its amendment Act No. 202/17), it is not necessary to obtain informed consent in fully anonymised retrospective studies on formalin-fixed paraffin-embedded tissues. At least one tissue block for each patient was retained in the archive to allow future clinical use such as re-evaluation or further studies.

## Results

### Tumor morphology

**Figure 1:**
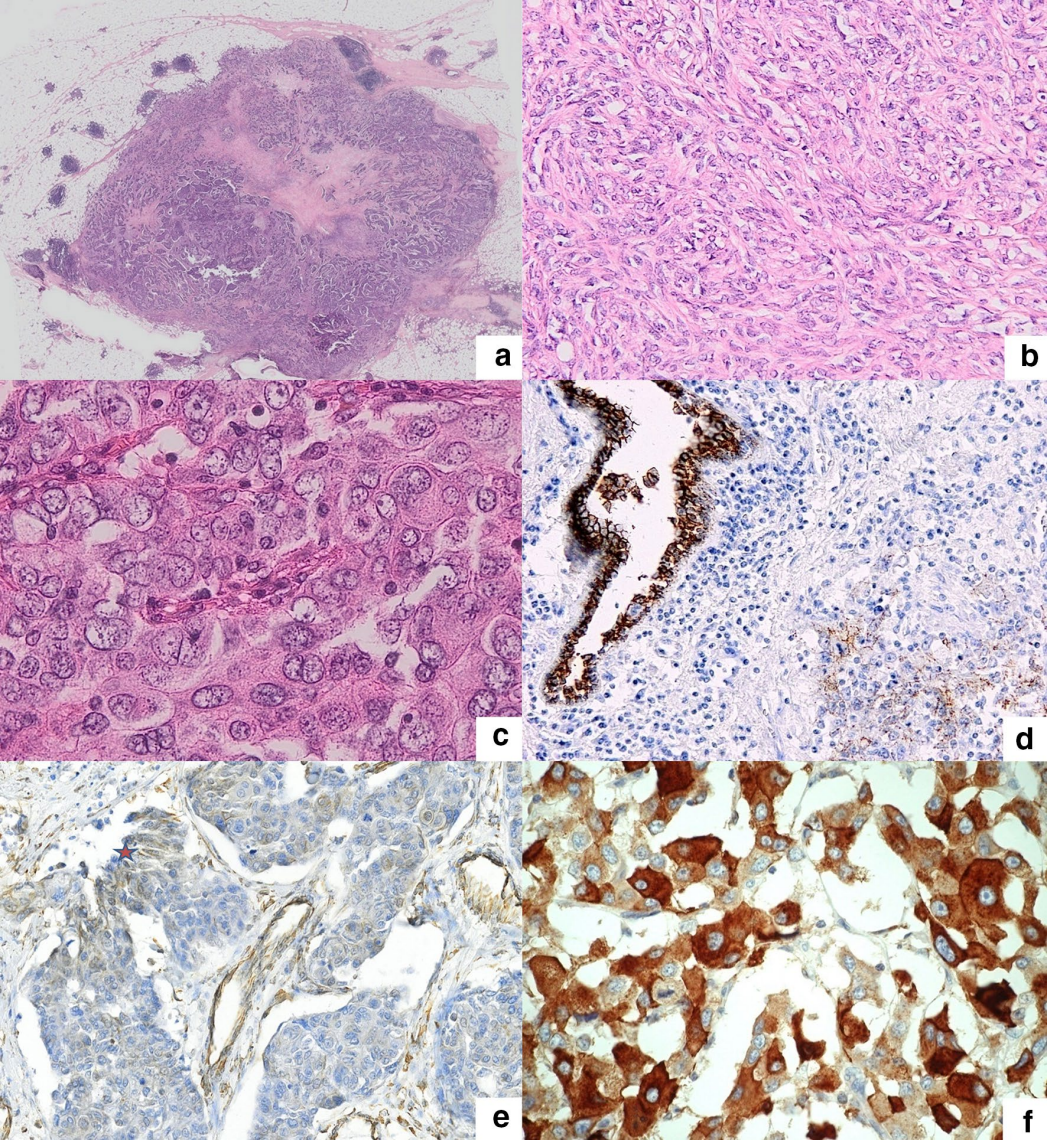
(**a**) Typical invasive carcinoma NST with medullary features—well defined and pushing tumor borders, syncitial architecture, central fibrosis and variable intensity of TILs (HE, magnification × 10). (**b**) Spindle—cell tumor transformation (HE, magnification × 200). (**c**) Apocrine tumor cell transformation (HE, magnification × 400). (**d**) Loss of E-cadherin expression in TNBC compared to normal tissue (magnification × 200). (**e**) Vimentin expression in tumor areas with spindle cell morphology (asterix; magnification × 200). (**f**) Areas of apocrine tumor cell transformation with corresponding expression of GCDFP-15 (magnification × 400).

The majority of tumors were classified as invasive carcinomas of no special type (NST) with medullary features (69/81; 85.2%). These tumors were predominantly characterized by well-defined and pushing borders and predominantly solid growth pattern with syncytial architecture (Fig. 1a). All TNBC samples displayed central necrosis or fibrosis. Additional tumors were histologically represented by pure invasive carcinomas NST (6/81; 7.4%), metaplastic carcinomas (5/81; 6.2%) and neuroendocrine (small cell) carcinoma (1/81; 1.2%). In situ carcinoma structures in the vicinity of main tumor area were found in more than one third of patients (32/81; 39.5%).

During detailed morphological analysis we observed a tendency to focal apocrine tumor cell transformation with characteristic voluminous, eosinophilic granular cytoplasm (37/81; 45.7%) and/or spindle cell transformation (33/81; 40.7%). Both spindle-cell and apocrine tumor cell transformations were detected in 23.5% (19/81) cases. Rarely, we also identified isolated foci of cribriform (5/81; 6.2%) or solitary tubular structures (11/81; 13.6%) or the presence of giant cells (6/81; 7.4%).

### Dysregulated miRNAs

**Figure 2:**
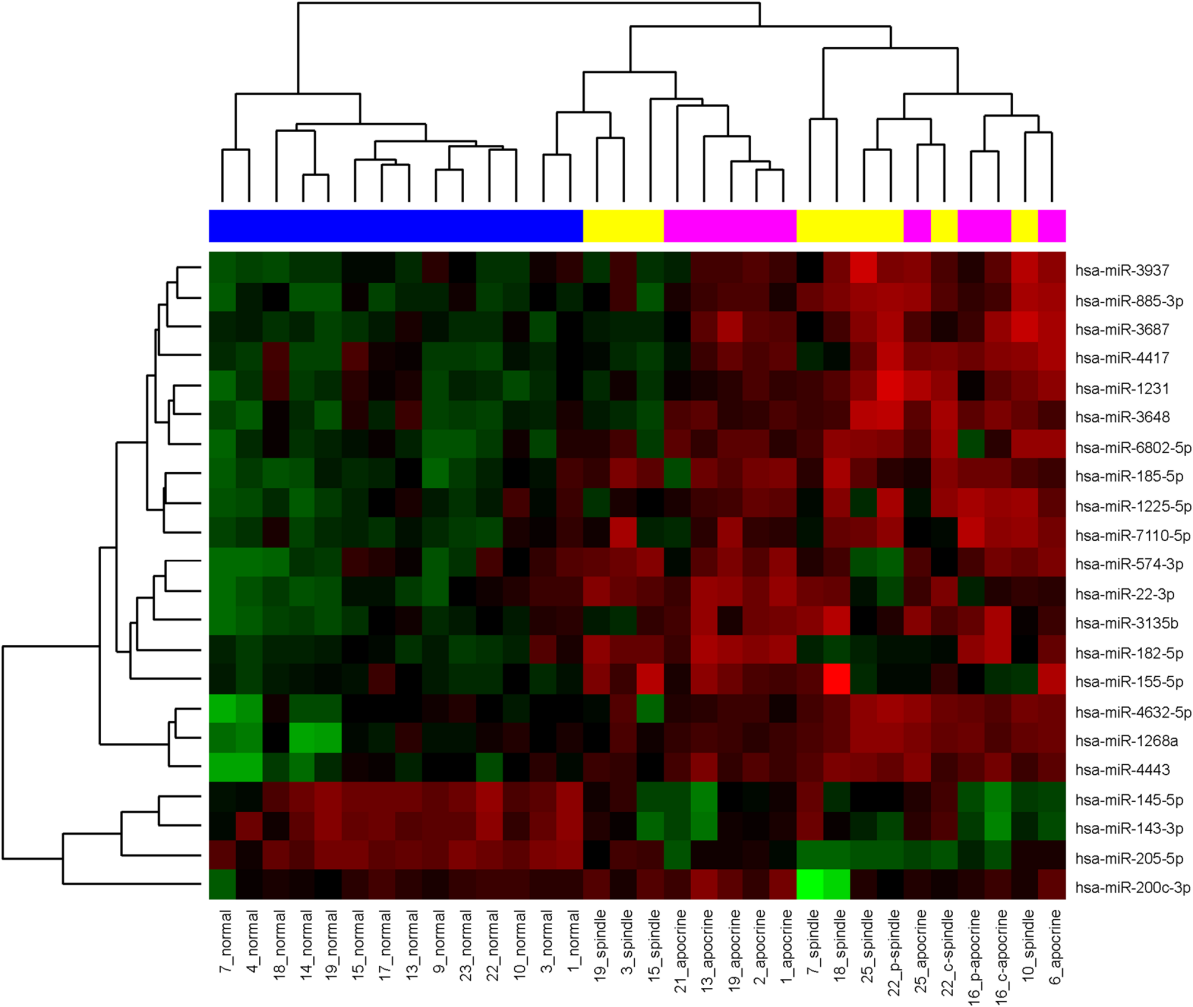
A heat map of a hierarchical clustering analysis of the microRNAs with differential expression between the normal, apocrine and spindle cell morphology. Red color indicates up-regulated miRNAs and green color indicates down-regulated mRNAs in cluster analysis. Blue, yellow and pink colors represent normal, spindle and apocrine cell morphology, respectively.

**Figure 3:**
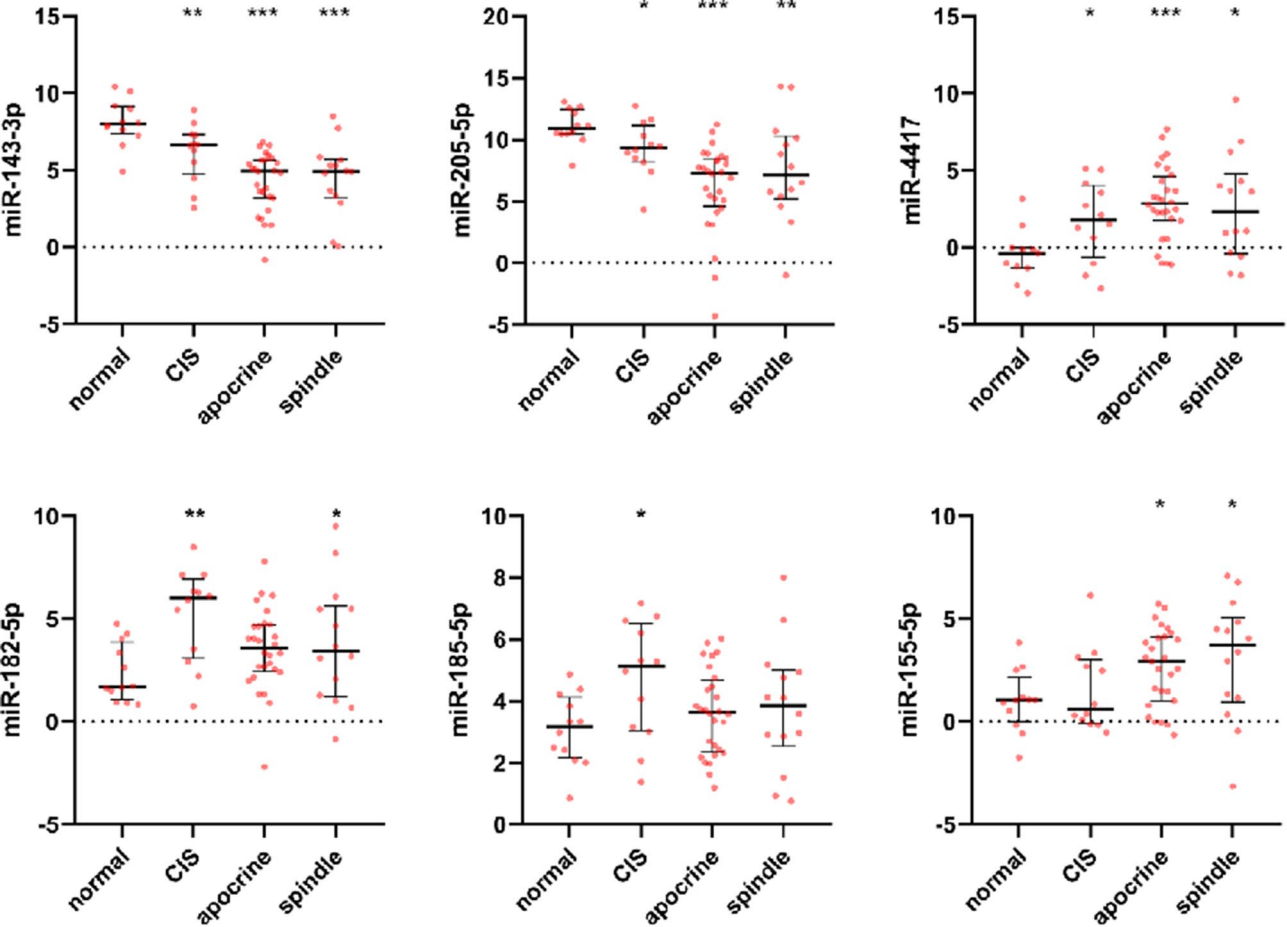
Expression analysis of selected miRNAs by RT-qPCR. Median and 25–75% percentiles are shown along with all values as dots. The figure displays 66 selected morphologies (normal—CIS—apocrine—spindle) out of 94 newly microdissected tissues from 70 TNBC patients (see also Supplementary Table 5). Relative miRNA expression is shown as an inverse value of ΔCt = Ct target miRNA –Ct reference U6. *P* values < 0.05, < 0.01 and < 0.001 are indicated by *, ** and ***, respectively.

We initially performed the laser capture microdissection of cells from areas with spindle-cell (Fig. 1b) and apocrine transformation (Fig. 1c), areas of dense TILs, ductal in situ carcinoma and epithelium of normal ducts and lobules on the set of 25 TNBCs with medullary features. After miRNA extraction and purification, we analyzed 74 microdissected samples by microarrays with 2578 human miRNAs. MiRNAs with the highest differential expression are listed in Table 3 (see also Supplementary Table 1a-c). The relation of miRNA expression profiles to specific tumor morphology and normal tissue is shown by heatmap (Fig. 2, Supplementary Fig. 1). Besides miRNAs from Table 3, hsa-miR-200c-3p and hsa-miR-155-5p were also included. Hsa-miR-200c-3p was the most downregulated miRNA in tumor areas with lymphocytic infiltration, while hsa-miR-155-5p was highly upregulated in these areas (Supplementary Table 1c). Hsa-miR-155–5-p was also upregulated in spindle cell morphology. We selected several candidate miRNAs for validation by RT-qPCR. First, the same RNA samples used for Affymetrix microarray analysis were measured by RT-qPCR for two miRNAs and results from both methods were highly correlated (Supplementary Fig. 2). Next, the localization of six miRNAs was verified by RT–qPCR on the extended set of of 94 newly microdissected tissue areas from 70 TNBC patients (Fig. 3, Supplementary Table 5). The best concordance with microarray analysis was observed for hsa-miR-143-3p, hsa-miR-205-5p and hsa-miR-4417, which also had highly significant differences in Table 3 (adjusted *p* values < 0.005). The other three miRNAs (hsa-miR-182-5p, hsa-miR-185-5p and hsa-miR-155-5p) had less significant differences both in the Affymetrix microarray and RT-qPCR analyses which is probably caused by higher variability of their expression.

**Table 4:** Available data on miRNAs involved in EMT and their target genes—oncogenic miRNAs.

Oncogenic miRNAs involved in EMT	Target	Results	References
miR-9	E-cadherin	±	^30^
miR-10a, miR-10b	PIK3CA , TGF-β1	−	^36–39^
miR-21	PTEN, PI3K, AKT, LZTFL1	±	^30^
miR-22-3p	FOXP1, HDAC4	+	^80^
miR-29a	SUV420H2	±	^45,79^
miR-30b -5p, miR-30d-5p,	KRT19, AKT2, CD24, TIMP1	± ; ±	^36–39^
miR-34a	TWIST1, CDK6	−	^59,60^
miR-93	PTEN, PI3K, AKT, AHNAK, TGFβR2	±	^49^
miR-99a-5p	KRT19, AKT2, CD24, TIMP1	±	^36–39^
miR – 100	SMARCA5, SMARCD1, BMPR2	±	^40^
miR-101	ZEB1/2, RHOA	±	^50^
miR-101-3p,	KRT19, AKT2, CD24, TIMP1	±	^36–39^
miR – 106b	PTEN, PI3K, AKT	±	^49^
miR-125b	APC, Wnt, β-catenin	−	^36–39^
miR-130a-3p	KRT19, AKT2, CD24, TIMP1	±	^36–39^
miR-155	C/EBP beta, RAD51, BRCA1, FOXO3A	+	^28^
miR-181a	Bim	±	^36–39^
miR-182-5p	PFN1, FOXF2	+	^36–39,47^
miR-192-5p,	KRT19, AKT2, CD24, TIMP1	±	^36–39^
miR-214-3p	KRT19, AKT2, CD24, TIMP2	±	^36–39^
miR-221	ADIPOR1, TRPS1, PTEN, ZEB2	± ; ±	^36–39,52,56^
miR-222	ADIPOR1, TRPS1, PTEN, ZEB2	± ; ±	^36–39,52,56^
miR-373	TXNIP, HIF1α, TWIST	±	^53^
miR-885-3p	B7-H3	+	^76^
miR-1225-5p	IRS1	+	^76^
miR-4443	TIMP2	+	^72^
miR-4800-3p	HNRNPA2/B1	±	^36–39^
miR-6836-3p	HNRNPA2/B1	±	^36–39^
miR-7110-5p	mTOR, RAS	+	^66^
miR-let-7f.-5p	KRT19, AKT2, CD24, TIMP1	−	^36–39^

+  = significant expression; ±  = non-significant expression; −  = non-changed expression; 0 = not analysed.

**Table 5:** Available data on miRNAs involved in EMT and their target genes—tumor suppressor miRNAs.

Tumor suppressor miRNAs involved in EMT	Target	Results	References
miR-7-5p	MMP2, ALDH1, MMP9, TWIST1, NOTCH1, AKT1	±	^66^
miR-15b	SUZ12, ZEB1/2, KLF4, BMI1	±	^24^
miR-17-5p,	MMP2, ALDH1, MMP9, TWIST1, NOTCH1, AKT1	−	^66^
miR-20b-5p	MMP2, ALDH1, MMP9, TWIST1, NOTCH1, AKT1	±	^66^
miR-26b-5p	MMP2, ALDH1, MMP9, TWIST1, NOTCH1, AKT1	−	^66^
miR-30a-5p	metadherin, twinfilin 1, vimentin, ROR1	±	^46^
miR-30c-5p	metadherin, twinfilin 1, vimentin, ROR1	±	^46^
miR-107	SUZ12, ZEB1/2, KLF4, BMI1	±	^24^
miR-128b,	SUZ12, ZEB1/2, KLF4, BMI1	±	^24^
miR-138-5p	RHBDD1	±	^24,36–39^
miR-143-5p	LIMK1	+	^77^
miR-145	SUZ12, ZEB1/2, KLF4, BMI2	+	^24^
miR-145-5p	EAPP, TGF-β	+	^76^
miR-146a-5p	MMP2, ALDH1, MMP9, TWIST1, NOTCH1, AKT1	±	^66^
miR-149-3p	MMP2, ALDH1, MMP9, TWIST1, NOTCH1, AKT2	±	^66^
miR-150-5p	MMP2, ALDH1, MMP9, TWIST1, NOTCH1, AKT3	±	^66^
miR-185-5p	S100A8/A9	+ ↑	^27^
miR-186-5p	MMP2, ALDH1, MMP9, TWIST1, NOTCH1, AKT4	−	^66^
miR-190	TGF-β, SMAD2	−	^24^
miR-200a, miR-200b, miR-200c	ZEB1/2, FN1, MSN, E-cadherin, vimentin	± ; ± ; ±	^24,36–39^
miR-200c/141 cluster	ZEB1/2, E-cadherin	0	^57^
miR-203	TGF-β, SNAI2	−	^48^
miR-205-5p	MMP2, ALDH1, MMP9, TWIST1, NOTCH1, AKT2	+	^66^
miR-206	MMP2, ALDH1, MMP9, TWIST1, NOTCH1, AKT3	−	^66^
miR-215	MMP2, ALDH1, MMP9, TWIST1, NOTCH1, AKT4	±	^66^
miR-223-3p	MMP2, ALDH1, MMP9, TWIST1, NOTCH1, AKT5	±	^66^
miR-335	CDH11, β-catenin	±	^53^
miR-339-5p	BLCAP	±	^22,36^
miR-363-5p	NOB1	−	^36–39,60^
miR-375	SHOX2, TGFβR1	−	^64^
miR-425	Lifr, Acvr1c, Pparγ	±	^65^
miR-516a-3p	Pygo2, Wnt	−	^67^
miR-520c, miR-520a-3p	NFκB, TGFβR2, IL8, CCND1, CD44	−	^36–39^
miR-574-3p	CLTC	+ ↑	^73,81^
miR-4417	TGF-β; SMAD2	+	^73^
miR-4655-3p	ERBB2	−	^76^
miR-4784	ERBB2	−	^76^
miR- let-7c	MMP2, ALDH1, MMP9, TWIST1, NOTCH1, AKT3	−	^66^

+  = significant expression; ±  = non-significant expression; −  = non-changed expression; 0 = not analyzed; ↑ = increased expression.

### Epithelial mesenchymal transition and pathway analysis

We also analyzed the available data concerning EMT-related miRNAs, their target genes and compared them with our results (see Tables 4, 5). The foci of spindle-cell morphology in breast cancer were found in 45.7% of cases. Apocrine cell transformation, considered to be a possible variant of EMT-related morphology, was recorded in 48.6% cases. Downregulation of E-cadherin and upregulation of vimentin were found also in our set of TNBC (Fig. 1d,e). Apocrine cell transformation was associated with increased expression of GCDF-15 (Fig. 1f).

Regarding candidates in Table 3, all three downregulated (hsa-miR-143-3p, hsa-miR-205-5p and hsa-miR-145-5p) and nine upregulated miRNAs (hsa-miR-22-3p, hsa-miR-182-5p, hsa-miR-185-5p, hsa-miR-574-3p, hsa-miR-885-3p, hsa-miR-1225-5p, hsa-miR-4417, hsa-miR-4443 and hsa-miR-7110-5p) have previously been reported in relation to EMT (Tables 4, 5). Novelly, we described a statistically significant increased expression of eight miRNAs without known importance in EMT (hsa-miR-1231, hsa-miR-1268a, hsa-miR-3135b, hsa-miR-3648, hsa-miR-3687, hsa-miR-3937, hsa-miR-4632-5p and hsa-miR-6802-5p). We have performed pathway analysis for these 8 miRNAs as well as for other upregulated (N = 9) and downregulated (N = 3) miRNAs from Table 3. Target genes of these miRNAs are provided as Supplementary Tables 2a-c. The results of pathway analyses are provided as Supplementary Tables 3a-c. Pathway analysis was also performed for the relevant groups of miRNAs and results are provided as Supplementary Table 4 and Supplementary Fig. 3. Although we cannot prove direct involvement of these miRNAs in the EMT process, there are several pathways (i.e. Wnt signaling, ErbB signaling, MAPK signaling, endocytosis and axon guidance) commonly affected by all these groups. The eight novel miRNAs might contribute to the cancer transformation and EMT process, but a more detailed mechanistic link will require additional functional experiments and investigation.

## Discussion

TNBCs are considered to be a morphologically and genetically heterogeneous molecular subtype of breast cancers with specific and variable response to chemotherapy^24^. The mechanism of arising chemoresistance lies in the induction of rare pre-existing subclones (adaptive resistance), new mutations (acquired resistance) or cancer-associated metabolic reprogramming (altered metabolism of glucose, lipids and amino acids)^25^. Our study provides detailed miRNA analysis of the unique set of chemotherapeutically non-influenced TNBCs from a morphological point of view.

MiRNAs are involved in many cancer–related pathways, such as DNA damage response, cell cycle, apoptosis, autophagy, tumor cell proliferation, migration and invasion or immune response, reported as the concept of cancer immunoediting^15, 26^. They are also associated with the patients’ overall survival and recurrence. Each miRNA has multiple targets which can be simultaneously modulated by several miRNAs. The mechanisms deregulating miRNA expression in TNBC involve genomic and epigenetic alterations (chromosomal abnormalities), defects of the genes in the miRNA biogenesis pathway (e.g. *Dicer, Drosha, TARBP2, XPO5*) and alterations of transcription factors regulating miRNA biological processes (e.g. SMAD, p53, ATM, c-Myc, E2F).

Our results demonstrate the importance of specific miRNAs in TNBC morphogenesis. We focused on the description and isolation of several forms of tumor cell differentiation, including apocrine and spindle cell morphology within individual TNBCs. These morphological changes are probably a manifestation of incipient EMT, corresponding to the specific changes in microRNA expression levels. With respect to EMT-related morphology, we proved a significant increased expression of hsa-miRNA-185-5p, hsa-miRNA-155-5p, hsa-miR-885-3p, hsa-miR-3687, considered as oncogenic miRNAs and down-regulation of tumor suppressive hsa-miR-205c-3p and hsa-miR-143-3p. However, the suppressive effect of miRNA-185-5p in breast cancer via regulation of S100A8/A9, nuclear factor-κB/Snail signaling pathway and programmed cell death was also reported in the literature^27^. miR-155 is a well-known miRNA with both oncogenic and tumor suppressive character. Hsa-miRNA-155-5p induces cell proliferation via activation of the STAT3 gene and reduces bufalin-induced apoptosis in TNBC cells^28^. On the other hand, this miRNA is highly expressed in immune cells, which may be related to better survival of TNBC patients due to higher presence of tumor infiltration lymphocytes^29,30^. The activation of CIAPIN1 protein which may depresshsa-miR-143-3p expression can be one of the potential mechanisms leading to therapeutic resistance^31^. Demethylation of the miR-200c promoter was found to be associated with tamoxifen reversed EMT and inhibition of cell migration in TNBCs^32^. Tumor suppressive miRNA-205-5p reduces TGF-β-induced EMT^33^. Its inhibition indicates a resistance to chemotherapy.

Recently published data indicate that EMT in breast cancer has typical morphological correlates such as changes of cellular shape and features resulting from spindle, giant and apocrine metaplasia^8^. Usually they are accompanied by changes of expression profile such as the downregulation of epithelial markers E-cadherin and cytokeratins as well as upregulation of mesenchymal markers N-cadherin, vimentin and transcription factor twist^18,34,35^. We also analyzed the available data on characteristic EMT-related miRNAs and compared them with our results^30,36–81^. In accordance with published data, our study showed the down-regulation of tumor suppressive hsa-miR-145-5p and hsa-miR-143-3p. Tumor suppressive EMT-related hsa-miR-145 promotes TNF-α-induced apoptosis through targeting cIAP1and may serve as a potential biomarker for an early diagnosis of TNBC. In our study, hsa-miRNA-205-5p was decreased in spindle cell and apocrine cell tumor morphologies. Increased expression of hsa-miR-182-5p in apocrine cell tumor morphology can confirm its oncogenic potential together with its relation to the EMT process. In relation to apocrine cell transformation we proved the significant increased expression of hsa-miR-182-5p, hsa-miR-4417, hsa-miR-3687, hsa-miR-1225-5p and down-regulation of hsa-miR-145-5p. We also found a significant profile for both spindle cell and apocrine tumor morphology which displayed simultaneous increased expressions of oncogenic hsa-miR-3135b, hsa-miR-3648, hsa-miR-4443, hsa-miR-7110-5p, hsa-miR-574-3p, hsa-miR-4632-5p, hsa-miR-22-3p, hsa-miR-1268a, hsa-miR-185-5p, hsa-miR-6802-5p, hsa-miR-885-3p and decreased expression of hsa-miR-143-3p and hsa-miR-205-5p.

We have also performed pathway analysis with the aim to elucidate the role of relevant miRNAs in EMT and other biological processes^82^. Although we cannot prove direct involvement of these miRNAs in the EMT process, there are several pathways (i.e. Wnt signaling, ErbB signaling, MAPK signaling, endocytosis and axon guidance) commonly affected by all these groups. The eight novel miRNAs might contribute to the cancer transformation and EMT process, but a more detailed mechanistic link will require additional functional experiments and investigation.

Among upregulated miRNAs, we have found two miRNAs (hsa-miR-3687 and hsa-miR-4417) which are not included in miRbase and assigned as non-confidential in TargetScan database. Nevertheless, several recent studies support their relevance by different methods. Benoist et al.^83^ identified hsa-miR-3687 as a novel prognostic marker for response in patients with castration-resistant prostate cancer treated with enzalutamide. This miRNA may also confer aggressiveness of oesophagus cancer through its target gene PGRMC2^84^. Using in-vitro and in-vivo models^85^ found that upregulation of hsa-miR-4417 and its target genes contribute to nickel chloride-promoted lung epithelial cell fibrogenesis and tumorigenesis. On the other hand, low expression of hsa-miR-4417 was significantly associated with worse prognosis in TNBC patients, while its overexpression was sufficient to inhibit migration and mammosphere formation of TNBC cells. We have observed upregulation of this miRNA both by microarray analysis and RT-qPCR in TNBC, in particular in apocrine cell morphology. Others also report increased expression of this miRNA in TNBC^86^. Further investigation is needed to clarify the validity of these two candidates which are currently not among confidentially identified miRNAs.

## Conclusions

Our findings indicate the importance of detailed description of morphological changes in TNBCs since they reflect miRNA expression which can be related to EMT as well as to prognosis and therapy resistance. Generally, it is shown that morphology of cells in cancers should be closely related to expression of specific miRNAs. This also demonstrate that previously detected heterogeneity of miRNAs in breast cancers needn´t be necessary accidental but it may be related to morphological character of cells. Understanding the function of miRNAs implicated in cancer pathogenesis, including the EMT process, also extends the range of new potential diagnostic and therapeutic options and can provide information on prognosis and response to therapy.

## Data availability

All important data generated or analyzed during this study are included in this article (and its Supplementary Information files). Raw data of miRNA expression profiling will be provided upon request.

## Supplementary Information

Supplementary Figure 1.

Supplementary Figure 2.

Supplementary Figure 3.

Supplementary Table 1a.

Supplementary Table 1b.

Supplementary Table 1c.

Supplementary Table 2a.

Supplementary Table 2b.

Supplementary Table 2c.

Supplementary Table 3a.

Supplementary Table 3b.

Supplementary Table 3c.

Supplementary Table 4.

Supplementary Table 5.
