# Supplementary figures and images for: Epithelial to mesenchymal transition and microRNA expression are associated with spindle and apocrine cell morphology in triple-negative breast cancer
Source: Sci Rep. 2021 Mar 4;11:5145. doi: 10.1038/s41598-021-84350-2 (PMC7933252; doi:10.1038/s41598-021-84350-2)

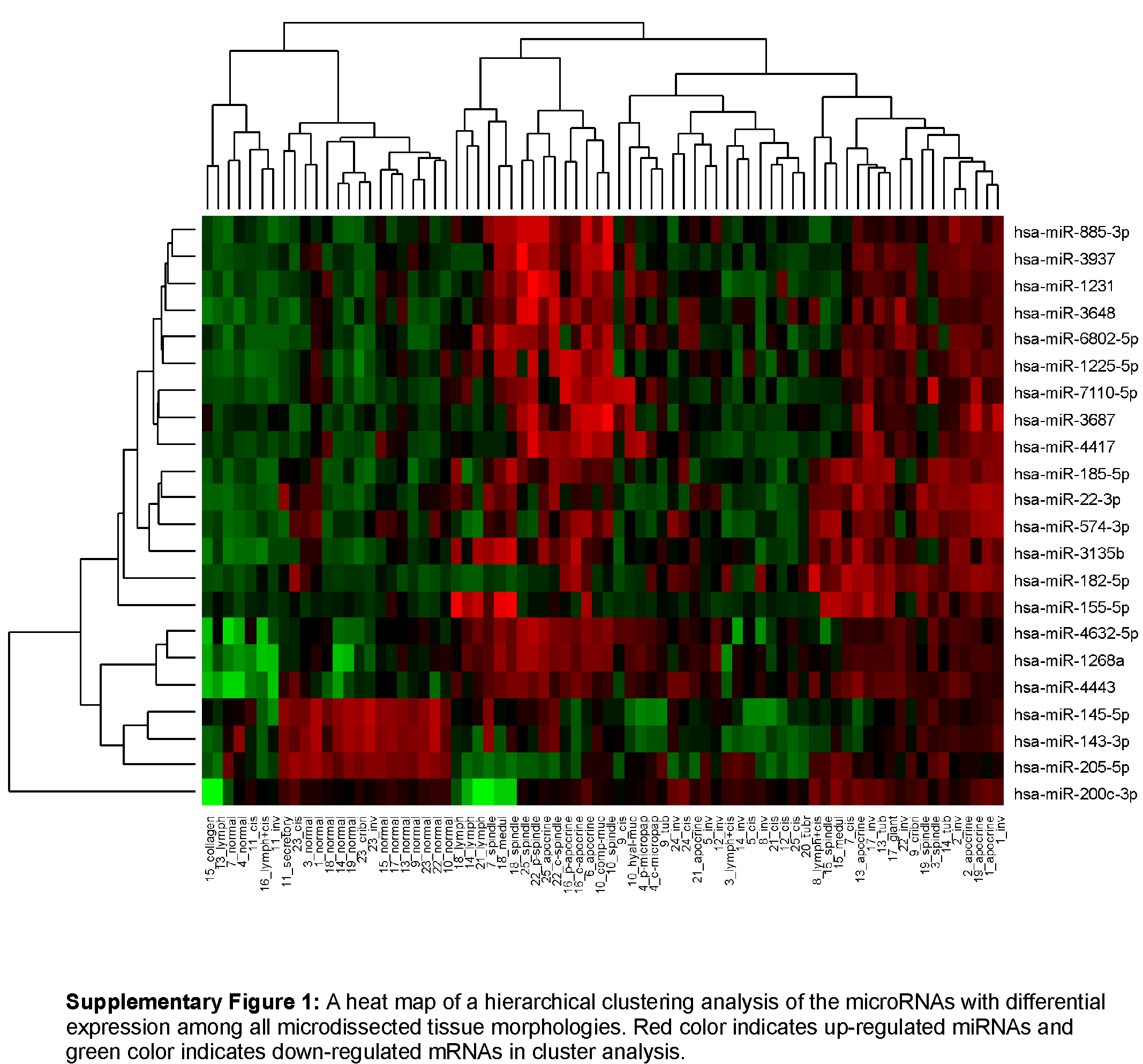

Supplement: Supplementary file 1 — Supplementary Figure 1. [file 41598_2021_84350_MOESM1_ESM.tif]

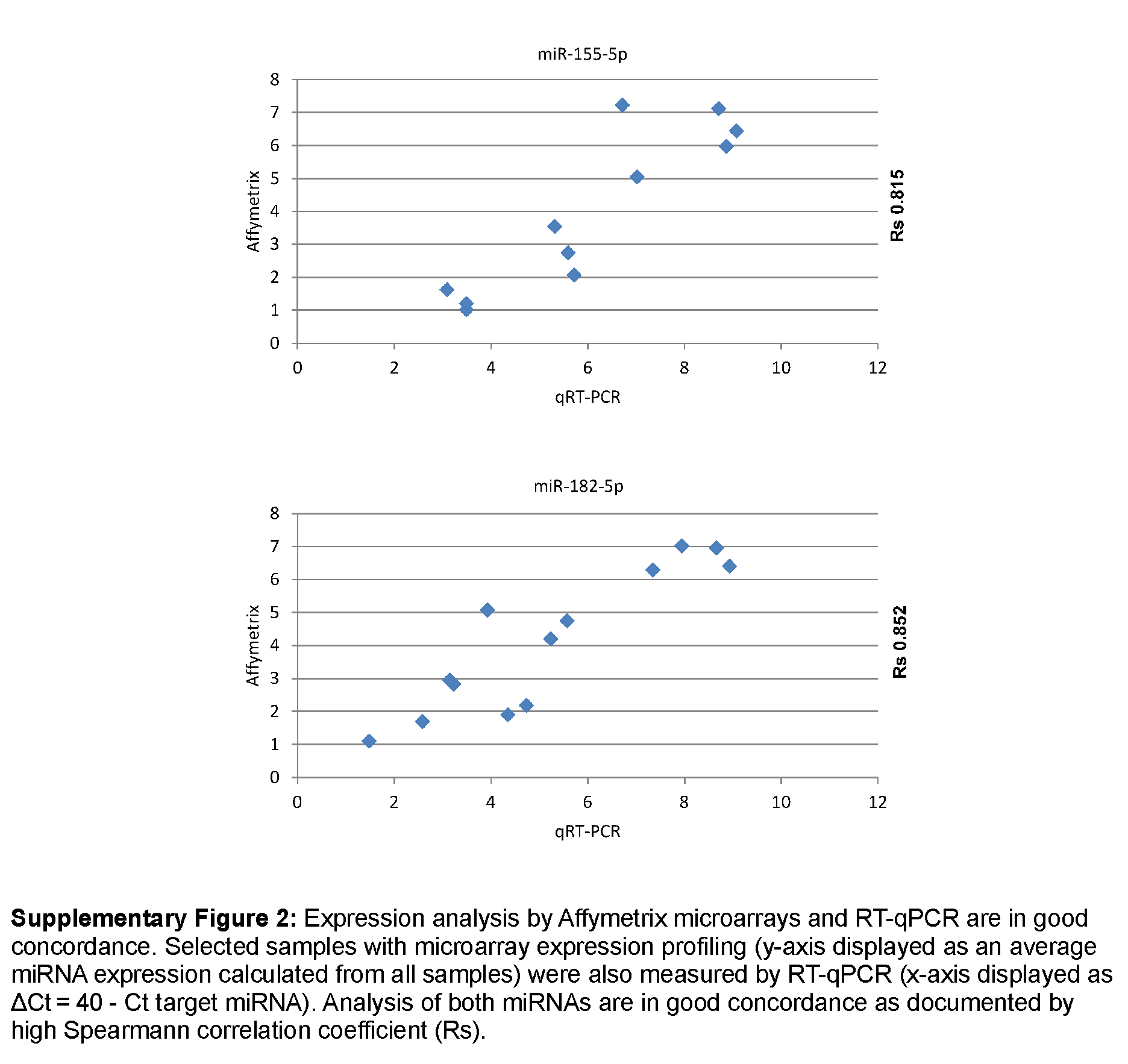

Supplement: Supplementary file 2 — Supplementary Figure 2. [file 41598_2021_84350_MOESM2_ESM.tif]

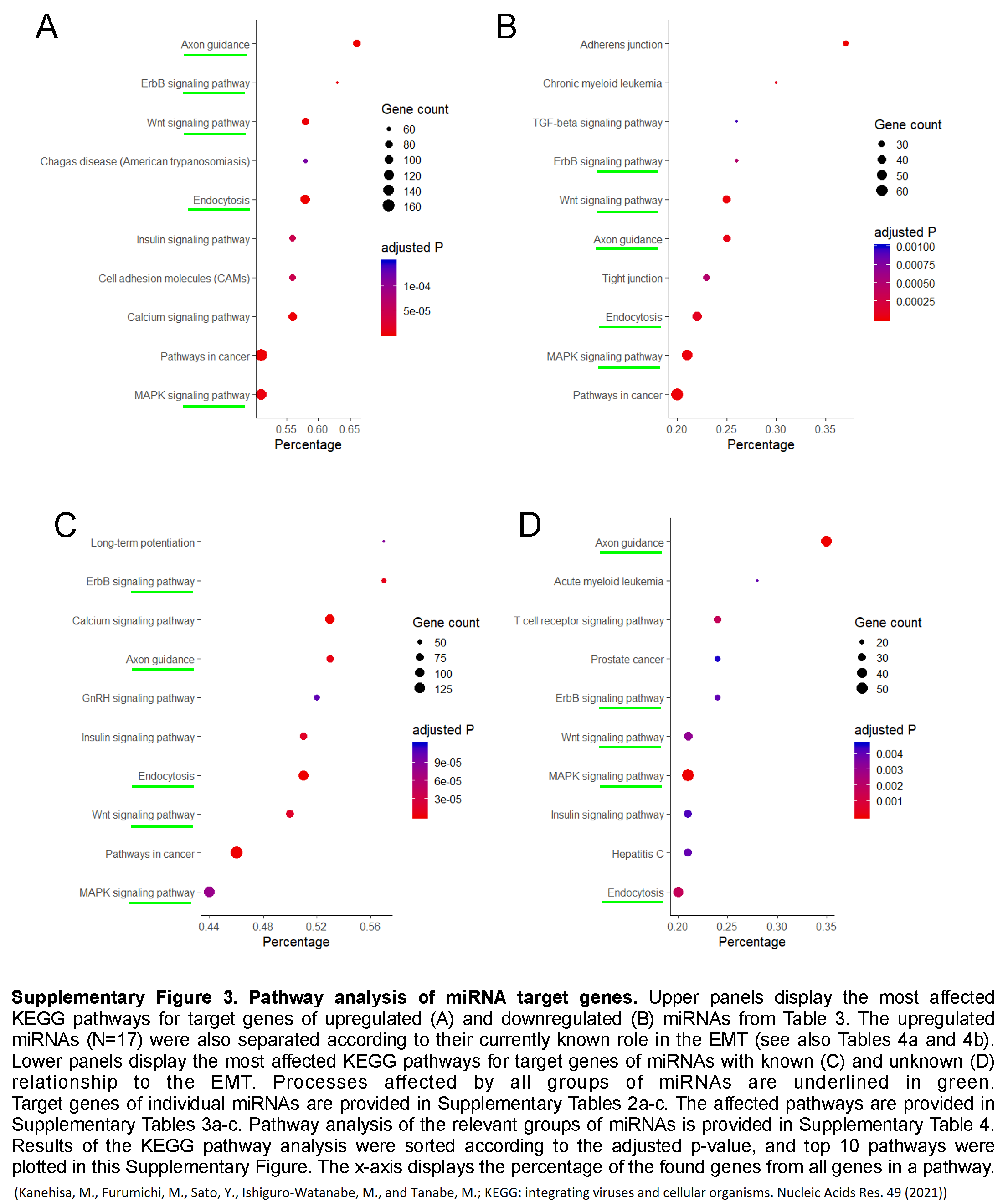

Supplement: Supplementary file 3 — Supplementary Figure 3. [file 41598_2021_84350_MOESM3_ESM.tif]
